# Risk factors associated with in-hospital mortality and a composite of major complications following paediatric congenital cardiac surgery: A systematic review protocol

**DOI:** 10.1371/journal.pone.0360693

**Published:** 2026-11-12

**Authors:** Palesa Mogane, Moses Kebalepile, Palesa Motshabi Chakane

**Affiliations:** Department of Anaesthesiology, School of Clinical Medicine, Faculty of Health Sciences, University of the Witwatersrand, Parktown, Johannesburg, South Africa; Ataturk University Faculty of Medicine, TÜRKIYE

## Abstract

**Background:**

Approximately 70% of patients born with congenital heart disease (CHD) require medical or surgical treatment in their first year of life. A reported 90–95% will survive to adulthood due to advances in paediatric cardiology, cardiac surgery and anaesthesia. These reported advances are still not available for over 90% of the children born with CHD in low-and middle-income countries (LMIC). The objectives of this study are to determine the pooled incidence of in-hospital all-cause mortality; identify risk factors associated with in-hospital all-cause mortality and a composite of major complications in paediatric patients undergoing congenital cardiac surgery; and to compare these risk factors between LMICs and high-income countries (HICs).

**Methods:**

A systematic review will be conducted in accordance with the Preferred Reporting Items for Systematic Reviews and Meta-Analyses (PRISMA) guidelines; this has been registered with PROSPERO 2024 (CRD42024618297). The Population, Exposure and Outcome (PEO) framework will guide the search strategy. Eligible studies will include prospective and retrospective cohort studies, case-control studies, interventional studies and registry/database studies reporting risk factors for in-hospital all-cause mortality and/or major postoperative complications in paediatric patients (≤18 years) undergoing cardiac surgery for CHD. A comprehensive electronic search of PubMed, Scopus, CINAHL, Cochrane Library and Web of Science will identify peer-reviewed English-language studies published from January 2000–2026. Two reviewers will independently screen studies, extract data using a standardised data extraction form, and assess risk of bias using the Newcastle-Ottawa Scale or ROBINS-I, as appropriate to study design. Where appropriate, we will perform random-effects meta-analysis to estimate pooled effect measures and 95% confidence intervals. Statistical heterogeneity will be assessed using the I² statistic, and results will otherwise be synthesised narratively. Where sufficient data are available, risk factors will be compared between LMICs and HICs.

**Discussion:**

In-hospital mortality and major complications following paediatric congenital cardiac surgery are associated with a combination of patient-, surgical- and perioperative risk factors. Identifying these facilitates early risk stratification and optimisation of modifiable risk factors to support targeted perioperative interventions and resource allocation, particularly in LMICs where evidence remains limited. A meta-analysis is not anticipated because of substantial heterogeneity in the study population.

## Background

Global cardiac surgery is defined as “an area for study, research, practice and advocacy that prioritises improving health outcomes and achieving health equity for all people worldwide who are affected by cardiac surgical conditions or have the need for cardiac surgical care” [1]. Congenital heart disease (CHD) affects 1 in 125 live births worldwide every year [2]. Approximately 70% of these patients require medical or surgical treatment in their first year of life [1]. A reported 90–95% survival to adulthood with long-term outcomes is feasible with advancements in paediatric cardiology, cardiac surgery and anaesthesia, access to prenatal screening, and early diagnosis and treatment [1]. These reported advances in care are still not available for over 90% of the children born with CHD in low- and middle-income countries (LMIC).

In an estimated population of one billion on the African continent, approximately 2500–3000 children undergo cardiac surgery annually [3], with South Africa and Egypt having better cardiac services than other countries on the continent [4]. South Africa operates on 40% of the population with CHD, with low access in the public sector [4], as evidenced by an estimated 50 public cardiac patients per million, as compared to 530 private patients per million, undergoing surgery annually in South Africa [4].

In Mozambique, Mocumbi et al. [5] reported late presentations among congenital cardiac patients, with 29% presenting with complications at the time of diagnosis. The operation rate was 36%, with only 26% of complex lesions operated on. In Rwanda, only 149 open cardiac procedures have been performed in the last 10 years by Team Heart, an international humanitarian effort [3]. In northern Uganda, the majority of their cases were operated on in Germany, the Cayman Islands, and India through funding from charity organisations [6].

Limited access to proper medical care has resulted in the mortality of almost one-third and half of children born with moderate to severe CHD in the neonatal period and early infancy, respectively [7].. Late presentation for surgery due to delayed referral or presentation, limited access to appropriate treatment, and a shortage of skilled experts and healthcare resources result in the progression of CHD and associated complications [8]. These include pulmonary hypertension (PHT), worsening cyanosis, and heart failure. Many children who survive to infancy and undergo surgery have debilitating postoperative outcomes [9]. In high-income countries (HIC), perioperative mortality rates (POMR) have decreased from 8.7% to less than 4% [10–13]. Anecdotal data from our centre suggest overall mortality rates of 10–12%, with 60–80% mortality among complex cardiac lesions, such as transposition of the great vessels.

Nonfatal complications are not adequately addressed, although they lead to morbidity in 96% of patients who survive to hospital discharge [14]. Subsequently, comprehensive outcome assessment should include both mortality and morbidity measures [14]. Multiple studies have defined a composite of major complications (morbidity) in varying ways. The selection of complications for this review was informed by established congenital cardiac surgery morbidity frameworks, including the Society of Thoracic Surgeons (STS)/Congenital Heart Surgeons Society (CHSS) definitions and the congenital heart surgery composite quality metric developed by Pasquali et al.[15], as well as subsequent multidisciplinary, patient- and family-informed work identifying the important postoperative morbidities [16]. These frameworks prioritise complications that represent life-threatening events, significant organ dysfunction or the need for major therapeutic intervention, and that have demonstrable implications for survival, length of hospital stay, healthcare resource utilisation, quality of life or longer-term outcomes. In addition, preference was given to complications with sufficiently objective and reproducible definitions to facilitate comparisons across studies.

Accordingly, the composite of major complications in this review includes cardiac arrest and low cardiac output syndrome, reflecting severe cardiovascular dysfunction; need for extracorpeal life support (ECLS), reflecting cardiorespiratory failure requiring advanced life support; prolonged pleural effusion; emergency reoperation and/or major haemorrhage requiring relook surgical re-exploration; multiorgan failure necessitating support (such as acute renal failure) reflecting severe systemic organ dysfunction; atrioventricular (AV) block requiring a permanent pacemaker; and new-onset neurological dysfunction persistent at discharge reflecting meaningful neurological morbidity [9,14,16]. Nosocomial infections and sepsis have been identified as common causes of mortality and morbidity in paediatric cardiac intensive care units but are often not included.

Reported rates of postoperative complications following paediatric congenital cardiac surgery vary substantially depending on the definition and severity of complications assessed, with rates ranging from 1.8% [10] to 84.1% [9]. Differences in the definition and measurement of complications limit comparisons between studies and support the need for a systematic evaluation of risk factors for major complications using clinically relevant and consistently defined outcomes.

Due to budgetary constraints, cardiac disease in the paediatric population is not a health priority in low- and middle-income countries (LMIC) [17]. Common infectious diseases are seen as more important and cost-effective than treating complex congenital and acquired cardiac disease [17]. Medical Research Council (MRC) statistics show that cardiac abnormalities are responsible for 1.2% of under 5-year mortality; the 8^th^ most important cause of mortality in South Africa [18]. This statistic is an underestimation of the actual numbers, as a significant number of children with undiagnosed cardiac disease present with upper respiratory infections, gastroenteritis, and sepsis with the cause unknown [17,18]. If left untreated, cardiac disease in the paediatric population can be burdensome on the healthcare system in South Africa.

Numerous studies have evaluated predictors of these outcomes; however, the evidence is fragmented and heterogeneous with respect to patient populations, cardiac diagnoses, surgical complexity, perioperative practices and definitions of adverse outcomes. This limits the ability to determine which risk factors are consistently associated with poor outcomes and whether established predictors are applicable across different healthcare settings. Furthermore, children undergoing cardiac surgery in LMICs may have different clinical and risk profiles from those in high-income countries because of differences in disease presentation, access to specialised care, delayed timing of referral, and availability of perioperative resources, which result in disease progression and an anticipated higher incidence of postoperative mortality and morbidity. A systematic review is therefore warranted to assess the available evidence on patient-, disease-, surgical-, anaesthetic- and other perioperative risk factors associated with in-hospital mortality and major complications following paediatric congenital cardiac surgery, to estimate the pooled incidence of mortality and to determine whether risk profiles differ according to country income setting. Such evidence may inform risk stratification, perioperative optimisation, clinical decision-making and resource allocation, particularly in resource-constrained settings.

There is currently no systematic review that looks at the risk factors associated with in-hospital mortality and a composite of major complications following congenital cardiac surgery. This research will be a constructive analysis of the published data on the subject. The ability to identify risk factors that contribute to complications enables early detection, analysis, and assessment of the likelihood of occurrence, review of impact, and minimisation of occurrence, thus favourably impacting outcomes [19]. Furthermore, this guides clinical decision-making and improves family preparation and counselling, resulting in improved perioperative management [20].

The systematic review aims to identify and describe risk factors (patient, surgical, anaesthetic and other) associated with in-hospital mortality and a composite of major complications in paediatric patients undergoing cardiac surgery for congenital cardiac disease. The primary objectives of this study are to determine the pooled incidence of in-hospital all-cause mortality; to identify risk factors associated with in-hospital all-cause mortality and a composite of major complications in paediatric patients presenting for cardiac surgery for congenital cardiac disease; and to compare these risk factors between low- and middle-income (developing) countries in comparison to high-income (developed) countries.

## Methods

### Eligibility criteria

A systematic literature review of existing literature will be performed to determine risk factors associated with in-hospital mortality and a composite of major complications following congenital cardiac surgery in the paediatric population. The systematic review has been registered with PROSPERO 2024 (CRD42024618297) and was developed using the guidelines set out by the Preferred Reporting Items for Systematic Reviews and Meta-Analyses (PRISMA) protocol (Table 1 in S1 File). There will be no patient involvement or potential harm; therefore, no consent is required. An ethics waiver was obtained from the University of Witwatersrand’s Human Research Ethics Committee (Medical) (M231004 M240523-A-0001).

Fig 1 shows a flow chart of literature screening for the systematic review. Data will be collected on the incidence and risk factors associated with in-hospital mortality and major complications in paediatric patients presenting for cardiac surgery using the Population, Exposure and Outcome (PEO) framework. Inclusion criteria will be as follows: retrospective and prospective cohort, case-control, and registry/database review studies, and grey literature published from 2000 to date; paediatric patients (<18 years of age) undergoing cardiac surgery for CHD; and in-hospital mortality and the presence of the composite of major complications will be the primary outcomes. Non-English literature studies and any duplicate studies will be excluded due to limited resources for translation. The period chosen for this study reflects the establishment of large multicentre databases examining congenital cardiac disease in the 2000s.

**Fig 1:**
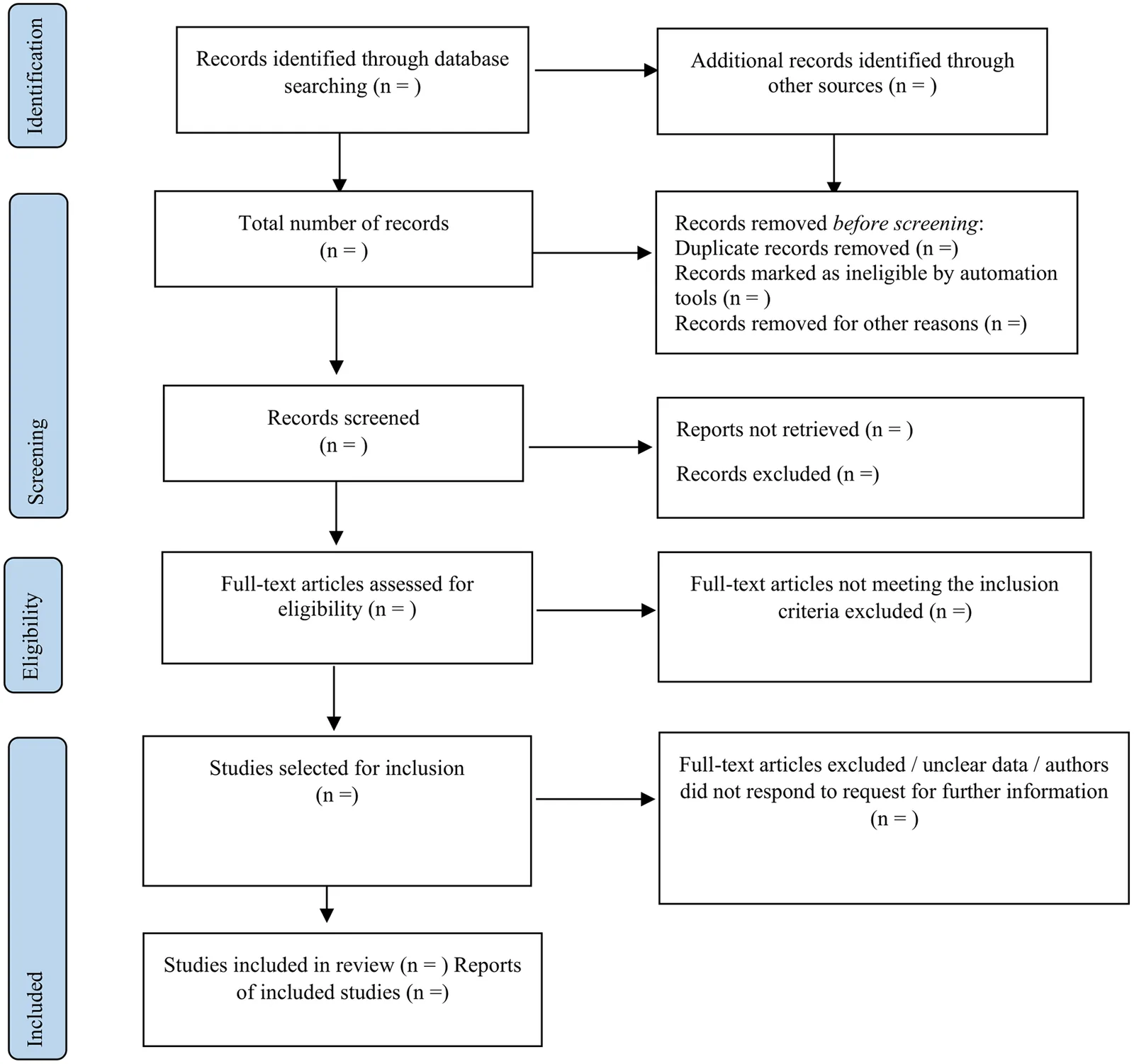
PRISMA flow chart of the literature screening process for the systematic review.

### Search strategy and selection process

The literature search will be performed on peer-reviewed English articles published in the following databases: PubMed, Scopus, CINAHL, Cochrane Library, and Web of Science. Important keywords and major medical subject headings (MESH terms) relevant to the topic will be used to extract data from the listed databases (Table 2 in S1 File). The PEO framework of the search strategies in the various databases is included (Table 3 in S1 File).

Furthermore, the abstracts and references for these articles will be screened for additional relevant articles. PM and GL will interrogate the full-text publications of all eligible studies to prepare for data extraction. Articles will then be screened independently for eligibility by myself (PM) and a second reviewer, GL, during August 2026.

### Data collection and extraction

Standardised data extraction and quality assessment sheets will be used to collect data from each eligible manuscript using EndNote ^TM^ 20 reference management tool. The COVIDENCE Systematic Review Software^®^ 2024 package (Veritas Health Innovation, Melbourne, Australia) will be used for article exportation, review, data extraction, and synthesis of results, which will be independently created by PM and GL. The following information will be collected: main author and date, country of study (HIC or LMIC), study population, study design, primary outcome (mortality or composite of complications), identified risk factors, and other relevant findings (Table 4 in S1 File). Any disputes will be reviewed and adjudicated by the third/fourth reviewers (KM/PMV).

Descriptive statistical methods, using frequencies and percentages, will be used to establish the most reported risk factors associated with in-hospital mortality and the composite of major complications. The outcome data will be presented as mean/standard mean differences and odds ratios. Pooled results, p-values, and 95% confidence intervals will be used to interpret differences in the importance of the data collected.

### Assessment of study quality and risk of bias

A full-text assessment of methodological quality and risk of bias will be undertaken independently for all eligible studies using tools appropriate to the study design. Observational cohort and case-control studies will be assessed using the Newcastle–Ottawa Scale (NOS) (Table 5 in S1 File), which evaluates study quality across the domains of selection, comparability, and ascertainment of exposure or outcome. Studies achieving a NOS score >7 will be considered to be of high methodological quality. Non-randomised intervention studies will be assessed using the Risk Of Bias In Non-randomized Studies of Interventions (ROBINS-I) tool, which evaluates risk of bias across domains including confounding, selection of participants, classification of interventions, deviations from intended interventions, missing data, measurement of outcomes, and selection of the reported result [21]. Disagreements between reviewers will be resolved through discussion and consultation with a third reviewer where necessary.

The Grading of Recommendations Assessment, Development and Evaluation (GRADE) guidelines will be used to assess the certainty of the overall body of evidence for each key outcome, considering risk of bias, inconsistency, indirectness, imprecision, and publication bias. Thus, the NOS and ROBINS-I will be used for study-level methodological quality and risk-of-bias assessment according to study design. In contrast, GRADE will be used to assess the certainty of cumulative evidence for each key outcome [22]. Where appropriate, publication bias and small study effects will be explored separately using funnel plots.

### Effect measures and data synthesis methods

Data will be extracted and synthesised from all studies meeting the eligibility criteria according to the predefined PEO framework. Risk factors will be categorised according to their temporal relationship to surgery as pre-, intra-, or postoperative factors. Preoperative risk factors will include patient- and disease-related characteristics present before surgery (e.g., age, nutritional status, genetic syndromes and baseline clinical status), while intra-operative and postoperative factors will include factors occurring during or after surgery (e.g., cardiopulmonary bypass time, cross-clamp time and postoperative complications). Where possible, risk factors will also be grouped according to clinically relevant domains to facilitate interpretation and minimize inappropriate pooling of heterogeneous variables.

For dichotomous outcomes, including in-hospital mortality and major complications, effect measures will include odds ratios (ORs), risk ratios (RRs), or hazard ratios (HRs) with corresponding 95% confidence intervals (CIs), as reported by the individual studies. Where studies report continuous risk factors, such as cardiopulmonary bypass or cross-clamp time, these will be synthesised using mean differences (MDs) or standardised mean differences (SMDs) with 95% CIs, as appropriate. Adjusted effect estimates will be preferentially used over unadjusted estimates where available. Statistical significance will be assessed using the reported p-values and 95% confidence intervals, with a p-value < 0.05 considered statistically significant.

Descriptive synthesis will be undertaken for all included studies, including study characteristics, sample size, population characteristics, risk factors evaluated, and reported outcome measures. The frequency with which individual risk factors are reported and their direction and magnitude of association with mortality or major complications will be summarised. Meta-analysis will be undertaken where studies are sufficiently homogeneous with respect to population, exposure/risk factor, outcome definition and effect measure. A random-effects model will be used as the primary approach because clinical and methodological heterogeneity between studies is anticipated. Where meta-analysis is not considered appropriate because of substantial heterogeneity, insufficient data, or differences in the definitions or measurement of risk factors or outcomes, findings will be synthesised narratively in accordance with the SWiM (Synthesis Without Meta-analysis) reporting guidance [23].

Statistical heterogeneity will be assessed using Cochran’s χ² (Q) test and the I² statistic, with I² values of approximately 25%, 50%, and 75% interpreted as low, moderate, and high heterogeneity, respectively. However, I² will be interpreted alongside the magnitude and direction of effect estimates, confidence intervals, and clinical and methodological heterogeneity between studies, rather than relying on predefined thresholds alone. Where appropriate, potential sources of heterogeneity will be explored through subgroup and/or sensitivity analyses, including by study design, geographical setting, patient characteristics, type of congenital cardiac surgery, and study quality/risk of bias.

For studies reporting prevalence estimates, a double-arcsine transformation may be applied where appropriate to stabilise variances and constrain confidence intervals within the 0–1 range before pooling. Pooled estimates will be presented with corresponding 95% CIs, together with measures of between-study heterogeneity.

Publication bias and small-study effects will be assessed where at least 10 studies are available for a given meta-analysis using funnel plots and, where appropriate, Egger’s regression test. The potential influence of individual studies on pooled estimates will be assessed using sensitivity analyses where appropriate. All statistical analyses will be performed using Stata statistical software, version 17 (StataCorp, College Station, TX, USA). Results will be presented using summary tables, forest plots, funnel plots where applicable, and flow diagrams.

### Study status and timeline

This manuscript represents a systematic review protocol submitted before completion of the review process. At the time of submission, the search strategy had been finalised, and preliminary database searches had commenced. Title and abstract screening is anticipated to be completed by September 2026, and full-text screening and data extraction are expected to be completed by October 2026. Final analysis, synthesis of results, and manuscript completion are anticipated by November 2026.

## Discussion

Surgical outcome is influenced by various factors, and understanding these factors is critical to minimising perioperative mortality and morbidity. Understanding these risk factors provides valuable insights into risk stratification, facilitating tailored care [24], planning, optimisation, resource allocation, perioperative care and quality improvement [25]. These risk factors include patient, anaesthetic, surgical and clinical factors.

Patient-related risk factors for in-hospital mortality and major complications have been identified as young age (particularly premature birth) and lower weight [9,12]. Greater surgical complexity and complex cardiac lesions, particularly univentricular and cyanotic lesions, are also associated with increased risk [9,12,26,27]. The presence of multiple cardiac defects and important comorbidities such as pulmonary hypertension, major chromosomal or syndromic diagnosis such as Down’s syndrome may further increase perioperative risk [9,12,27]. Extracardiac anomalies have also been incorporated into established approaches to congenital cardiac surgical risk stratification [26,27]. Preoperative malnutrition is associated with increased mortality and adverse postoperative outcomes [28]. A lower neutrophil-to-lymphocyte ratio has been reported among children who do not survive [29]. This may speak to sepsis and surgical site infections that have been shown to contribute to outcomes in LMICs [30]. Although children in LMICs are at a higher risk of having most of these risk factors, existing data on the subject and postoperative mortality or complications involving them, especially in African patients, are limited (see Table 6 in S1 File).

Surgical-related risk factors that have been associated with outcome are increased surgical complexity (higher categories of RACHS-1, especially 4 and 5, or a higher ABC score), longer cardiopulmonary bypass (CPB) duration, longer aortic cross-clamp (AXC) and circulatory arrest time, delayed sternal closure, emergency procedures, and multiple surgical procedures during primary admission [19,31]. Other-related risk factors include elevated postoperative lactate, glucose, and CK levels; low median central venous oxygen saturations; higher median central venous oxygen saturations; preoperative ventilation and intensive care unit (ICU) staffing/hospital resources [31]..

### Limitations

This review may be limited by restriction of the literature search to English-language publications, which may introduce language bias. Substantial clinical and methodological heterogeneity is anticipated, including differences in patient characteristics, congenital cardiac diagnoses, surgical complexity, perioperative management, healthcare settings, study design, and definitions and identification of mortality and major complications. Variation in the definition and composition of major complication outcomes may limit comparability between studies and the feasibility of meta-analysis. Differences in adjustment for confounding and incomplete reporting of risk factors or effect estimates may further affect the synthesis of associations. Publication bias and selective outcome reporting may also occur, particularly where studies with significant findings are preferentially published. Temporal changes in surgical techniques, perioperative care and institutional resources may additionally contribute to variation in outcomes and limit generalisability across settings. These limitations will be considered when interpreting the findings and assessing the certainty of the evidence.

## Conclusion

Careful patient selection and medical optimization of patients aligned with specific expertise at dedicated children’s hospitals may lead to improvement in perioperative mortality rates in paediatric patients with congenital heart disease presenting for cardiac surgery. Early identification and intervention targeting these risk factors may improve postoperative outcomes and reduce complications in this vulnerable population; however, large, prospective, multicenter studies are needed to further investigate the predictors of mortality following surgery for CHDs.

## Supporting information

S1 File Supporting information containing Table S1-S6. (DOCX)

## Abbreviations

CHD: Congenital heart disease
LMIC: Low- and middle-income countries
HIC: High-income countries
PHT: Pulmonary hypertension
POMR: Perioperative mortality rate
ECLS: Extracorporeal life support
AV: Atrioventricular
ICU: Intensive care unit
GCS: Global Cardiac Surgery
RACHS-1: Risk adjustment for congenital surgery score
ABC: Aristotle basic complexity score
CPB: Cardiopulmonary bypass
AXC: Aortic cross-clamp.
