# Supplementary material for: Risk factors associated with in-hospital mortality and a composite of major complications following paediatric congenital cardiac surgery: A systematic review protocol
Source: PLoS One. 2026 Nov 12;21(11):e0360693. doi: 10.1371/journal.pone.0360693 (PMC13759102; doi:10.1371/journal.pone.0360693)
Supplement: Supplementary file 1 [file pone.0360693.s001.docx]

**Supporting Information**

**Table 1. PRISMA checklist 2020**

| **Section and Topic** | **Item #** | **Checklist item** |
| --- | --- | --- |
| **TITLE** | | |
| Title | 1 | Identify the report as a systematic review. |
| **ABSTRACT** | | |
| Abstract | 2 | See the PRISMA 2020 for Abstracts checklist. |
| **INTRODUCTION** | | |
| Rationale | 3 | Describe the rationale for the review in the context of existing knowledge. |
| Objectives | 4 | Provide an explicit statement of the objective(s) or question(s) the review addresses. |
| **METHODS** | | |
| Eligibility criteria | 5 | Specify the inclusion and exclusion criteria for the review and how studies were grouped for the syntheses. |
| Information sources | 6 | Specify all databases, registers, websites, organisations, reference lists and other sources searched or consulted to identify studies. Specify the date when each source was last searched or consulted. |
| Search strategy | 7 | Present the full search strategies for all databases, registers and websites, including any filters and limits used. |
| Selection process | 8 | Specify the methods used to decide whether a study met the inclusion criteria of the review, including how many reviewers screened each record and each report retrieved, whether they worked independently, and if applicable, details of automation tools used in the process. |
| Data collection process | 9 | Specify the methods used to collect data from reports, including how many reviewers collected data from each report, whether they worked independently, any processes for obtaining or confirming data from study investigators, and if applicable, details of automation tools used in the process. |
| Data items | 10a | List and define all outcomes for which data were sought. Specify whether all results that were compatible with each outcome domain in each study were sought (e.g. for all measures, time points, analyses), and if not, the methods used to decide which results to collect. |
|  | 10b | List and define all other variables for which data were sought (e.g. participant and intervention characteristics, funding sources). Describe any assumptions made about any missing or unclear information. |
| Study risk of bias assessment | 11 | Specify the methods used to assess risk of bias in the included studies, including details of the tool(s) used, how many reviewers assessed each study and whether they worked independently, and if applicable, details of automation tools used in the process. |
| Effect measures | 12 | Specify for each outcome the effect measure(s) (e.g. risk ratio, mean difference) used in the synthesis or presentation of results. |
| Synthesis methods | 13a | Describe the processes used to decide which studies were eligible for each synthesis (e.g. tabulating the study intervention characteristics and comparing against the planned groups for each synthesis (item #5)). |
|  | 13b | Describe any methods required to prepare the data for presentation or synthesis, such as handling of missing summary statistics, or data conversions. |
|  | 13c | Describe any methods used to tabulate or visually display results of individual studies and syntheses. |
|  | 13d | Describe any methods used to synthesize results and provide a rationale for the choice(s). If meta-analysis was performed, describe the model(s), method(s) to identify the presence and extent of statistical heterogeneity, and software package(s) used. |
|  | 13e | Describe any methods used to explore possible causes of heterogeneity among study results (e.g. subgroup analysis, meta-regression). |
|  | 13f | Describe any sensitivity analyses conducted to assess robustness of the synthesized results. |
| Reporting bias assessment | 14 | Describe any methods used to assess risk of bias due to missing results in a synthesis (arising from reporting biases). |
| Certainty assessment | 15 | Describe any methods used to assess certainty (or confidence) in the body of evidence for an outcome. |
| **RESULTS** | | |
| Study selection | 16a | Describe the results of the search and selection process, from the number of records identified in the search to the number of studies included in the review, ideally using a flow diagram. |
|  | 16b | Cite studies that might appear to meet the inclusion criteria, but which were excluded, and explain why they were excluded. |
| Study characteristics | 17 | Cite each included study and present its characteristics. |
| Risk of bias in studies | 18 | Present assessments of risk of bias for each included study. |
| Results of individual studies | 19 | For all outcomes, present, for each study: (a) summary statistics for each group (where appropriate) and (b) an effect estimate and its precision (e.g. confidence/credible interval), ideally using structured tables or plots. |
| Results of syntheses | 20a | For each synthesis, briefly summarise the characteristics and risk of bias among contributing studies. |
|  | 20b | Present results of all statistical syntheses conducted. If meta-analysis was done, present for each the summary estimate and its precision (e.g. confidence/credible interval) and measures of statistical heterogeneity. If comparing groups, describe the direction of the effect. |
|  | 20c | Present results of all investigations of possible causes of heterogeneity among study results. |
|  | 20d | Present results of all sensitivity analyses conducted to assess the robustness of the synthesized results. |
| Reporting biases | 21 | Present assessments of risk of bias due to missing results (arising from reporting biases) for each synthesis assessed. |
| Certainty of evidence | 22 | Present assessments of certainty (or confidence) in the body of evidence for each outcome assessed. |
| **DISCUSSION** | | |
| Discussion | 23a | Provide a general interpretation of the results in the context of other evidence. |
|  | 23b | Discuss any limitations of the evidence included in the review. |
|  | 23c | Discuss any limitations of the review processes used. |
|  | 23d | Discuss implications of the results for practice, policy, and future research. |
| **OTHER INFORMATION** | | |
| Registration and protocol | 24a | Provide registration information for the review, including register name and registration number, or state that the review was not registered. |
|  | 24b | Indicate where the review protocol can be accessed, or state that a protocol was not prepared. |
|  | 24c | Describe and explain any amendments to information provided at registration or in the protocol. |
| Support | 25 | Describe sources of financial or non-financial support for the review, and the role of the funders or sponsors in the review. |
| Competing interests | 26 | Declare any competing interests of review authors. |
| Availability of data, code and other materials | 27 | Report which of the following are publicly available and where they can be found: template data collection forms; data extracted from included studies; data used for all analyses; analytic code; any other materials used in the review. |

*From:* Page MJ, McKenzie JE, Bossuyt PM, Boutron I, Hoffmann TC, Mulrow CD, et al. The PRISMA 2020 statement: an updated guideline for reporting systematic reviews. BMJ 2021;372:n71. doi: 10.1136/bmj.n71. This work is licensed under CC BY 4.0. To view a copy of this license, visit <https://creativecommons.org/licenses/by/4.0/>

**Table 2. Keywords and major medical subject headings (MESH terms) used in databases**

| **Query** | **MeSH term/Phrases** |
| --- | --- |
| **POPULATION/PROBLEM**:  Paediatric | Paediatric* [Title/Abstract], Pediatrics [MeSH], Pediatric* [Title/Abstract], Child* [Title/Abstract], Child [MeSH], Infant* [Title/Abstract], Neonat* [Title/Abstract], Newborn* [Title/Abstract], Adolescen* [Title/Abstract] |
| Cardiac | Congenital cardiac surgery [MeSH], Congenital cardiac surgery [Title/Abstract], Congenital heart surgery[Title/abstract] |
| Post surgery | "postoperative period"[MeSH Terms] OR "postoperative"[Title/Abstract] OR "perioperative"[Title/Abstract] OR "following surger*"[Title/Abstract] OR "after surger*"[Title/Abstract] OR "post surger*"[Title/Abstract] |
| **EXPOSURE:**  Risk factors | “Risk factor*” [Title/Abstract], Risk factors [MeSH], Risk* [Title/Abstract], Hazard* [Title/Abstract], Odds [Title/Abstract], Predict* [Title/Abstract], Likel* [Title/Abstract], Associat* [Title/Abstract], High* [Title/Abstract], Increas* [Title/Abstract], Factor* [Title/Abstract]  Predictors |
| **OUTCOME:** | ("hospital mortality"[MeSH Terms] AND "in hospital mortalit*"[Title/Abstract] AND "in hospital death*"[Title/Abstract]) OR "mortality"[MeSH Terms] OR "mortalit*"[Title/Abstract] OR "morbidit*"[Title/Abstract] OR "complication*"[Title/Abstract] OR "adverse event*"[Title/Abstract] OR "adverse outcome*"[Title/Abstract] OR "survival"[Title/Abstract] OR "outcome*"[Title/Abstract] |
| Composite of major complications | Cardiac arrest  Arythmia  Permanent pacemaker  Mechanical circulatory support, VAD, ECMO  Neurological deficit, dysfunction, injury  Renal dysfunction, disease, failure, injury  Dialysis, renal replacement therapy  Emergency surgery |

† Scopus search was performed using the keywords from the [Title/Abstract] searches of the PubMed strategy

**Table 3. Database search strategy according to PEO framework**

| **Database** | **PEO framework** | **MeSH terms/phrases** |
| --- | --- | --- |
| **PubMed/**  **Medline** | **Population** | "paediatric*"[All Fields] OR "paediatrics"[All Fields] OR "pediatrics"[MeSH Terms] OR "pediatrics"[All Fields] OR "paediatric"[All Fields] OR "pediatric"[All Fields] OR "child*"[All Fields] OR "infant*"[All Fields] OR "neonate*"[All Fields]  AND  "congenital cardiac surger*"[All Fields] OR "congenital heart surger*"[All Fields]  AND  "postoperative period"[All Fields] OR "perioperative period"[All Fields] OR ("postoperative period"[MeSH Terms] OR ("postoperative"[All Fields] AND "period"[All Fields]) OR "postoperative period"[All Fields] OR "postop"[All Fields] OR "postoperative"[All Fields] OR "postoperatively"[All Fields] OR "postoperatives"[All Fields]) OR ("perioperative"[All Fields] OR "perioperatively"[All Fields]) OR "after surger*"[All Fields] OR "following surger*"[All Fields] |
|  | **Exposure of interest** | "risk factor*"[All Fields] OR "predict*"[All Fields] OR "risk*"[All Fields] OR “contributor*”[All Fields] OR “determinant*”[All Field] OR “associate*”[All Fields] |
|  | **Outcomes** | "in hospital mortalit*"[All Fields] OR "mortalit*"[All Fields] OR "morbidit*"[All Fields] OR "complication*"[All Fields] OR “adverse event*”[All Fields] OR “adverse outcome*”[All Fields] OR “outcome*” |
| **Scopus** | ( TITLE-ABS KEY( paediatric* OR pediatric* OR child* OR infant* OR neonat*)  AND TITLE-ABS-KEY ( "congenital heart surger*" OR "congenital cardiac surger*" ) AND TITLE-ABS-KEY ( "risk factor*" OR risk* OR predict* ) AND TITLE-ABS-KEY ( postoperative OR perioperative OR "following surger*" OR "after surger*" ) AND TITLE-ABS-KEY ( "in hospital mortalit*" OR mortalit* OR morbidit* OR complication* OR "composite complication*" ) ) AND ( LIMIT-TO ( EXACTKEYWORD , "Human" ) ) AND PUBYEAR > 1999 AND PUBYEAR < 2027 | |
| **CINAHL** | (pediatric* or paediatric* or child* or infant* or neonat*) AND ("congenital cardiac surger*" or "congenital heart surger*" or "heart defect*" or "cardiac defect*") AND (risk or "risk factor*" or predict*) AND (perioperative or postoperative or "after surg*" or "following surg*") AND ("in hospital mortalit*" OR mortalit* OR morbidit* OR complication*) | |
| **Cochrane Library/ Web of Science** | paediatric* OR pediatric* OR child* OR infant* OR neonat* (All Fields) and "congenital heart surger*" OR "congenital cardiac surger*" (All Fields) and "risk factor*" OR risk* OR predict* (All Fields) and postoperative OR perioperative OR "following surger*" OR "after surger*" (All Fields) and "in hospital mortalit*" OR mortalit* OR morbidit* OR complication* OR "composite complication*" (All Fields) | |
| **EMBASE** | Not available at institution. | |

*****Searches in all databases will be limited to paediatric (<18 yrs), human, 2000-2026

**Table 4.** **Data collection template showing characteristics of included studies**

**Author details:**

- Names
- Country/region
- LMIC vs HIC (according to World Bank income classification)
- Institution
- Email address

**Study period:**

- Start date and end date

**Study design:**

- Single/multicentre
- Retro/prospective
- Observational/interventional study
- Observational – cohort/case-control/cross-sectional/longitudinal study
- Interventional study

**Inclusion and exclusion criteria**

**Study population characteristics:**

- Age, sex
- Participant selection/diagnosis
- Sample size
- Surgical procedure/intervention

**Outcome:**

- Mortality and/or composite major complication
- Incidence

**Risk factor identified** – mean (SD), median (IQR), UOR (CI), p-value

- Preoperative (patient factors)
- Intra- or postoperative (surgical, anaesthetic, other)
- Postoperative (clinical factors/biomarkers)

**Risk of bias assessment** (NOS or ROBIN-I)

**GRADE assessment**

**Table 5. Risk of bias tool - Newcastle-Ottawa Quality Assessment Scale**

**NEWCASTLE - OTTAWA QUALITY ASSESSMENT SCALE**

**CASE CONTROL STUDIES**

Note: A study can be awarded a maximum of one star for each numbered item within the Selection and Exposure categories. A maximum of two stars can be given for Comparability.

**Selection**

1) Is the case definition adequate?

a) yes, with independent validation **🟑**

b) yes, eg record linkage or based on self reports

c) no description

2) Representativeness of the cases

a) consecutive or obviously representative series of cases **🟑**

b) potential for selection biases or not stated

3) Selection of Controls

a) community controls **🟑**

b) hospital controls

c) no description

4) Definition of Controls

a) no history of disease (endpoint) **🟑**

b) no description of source

**Comparability**

1) Comparability of cases and controls on the basis of the design or analysis

a) study controls for _______________ (Select the most important factor.) **🟑**

b) study controls for any additional factor **🟑** (This criteria could be modified to indicate specific control for a second important factor.)

**Exposure**

1) Ascertainment of exposure

a) secure record (eg surgical records) **🟑**

b) structured interview where blind to case/control status **🟑**

c) interview not blinded to case/control status

d) written self report or medical record only

e) no description

2) Same method of ascertainment for cases and controls

a) yes **🟑**

b) no

3) Non-Response rate

a) same rate for both groups **🟑**

b) non respondents described

c) rate different and no designation

**NEWCASTLE - OTTAWA QUALITY ASSESSMENT SCALE**

**COHORT STUDIES**

Note: A study can be awarded a maximum of one star for each numbered item within the Selection and Outcome categories. A maximum of two stars can be given for Comparability

**Selection**

1) Representativeness of the exposed cohort

a) truly representative of the average _______________ (describe) in the community **🟑**

b) somewhat representative of the average ______________ in the community **🟑**

c) selected group of users eg nurses, volunteers

d) no description of the derivation of the cohort

2) Selection of the non exposed cohort

a) drawn from the same community as the exposed cohort **🟑**

b) drawn from a different source

c) no description of the derivation of the non exposed cohort

3) Ascertainment of exposure

a) secure record (eg surgical records) **🟑**

b) structured interview **🟑**

c) written self report

d) no description

4) Demonstration that outcome of interest was not present at start of study

a) yes **🟑**

b) no

**Comparability**

1) Comparability of cohorts on the basis of the design or analysis

a) study controls for _____________ (select the most important factor) **🟑**

b) study controls for any additional factor **🟑** (This criteria could be modified to indicate specific control for a second important factor.)

**Outcome**

1) Assessment of outcome

a) independent blind assessment **🟑**

b) record linkage **🟑**

c) self report

d) no description

2) Was follow-up long enough for outcomes to occur

a) yes (select an adequate follow up period for outcome of interest) **🟑**

b) no

3) Adequacy of follow up of cohorts

a) complete follow up - all subjects accounted for **🟑**

b) subjects lost to follow up unlikely to introduce bias - small number lost - > ____ % (select an adequate %) follow up, or description provided of those lost) **🟑**

c) follow up rate < ____% (select an adequate %) and no description of those lost

d) no statement

**Table 6. Summary of referenced literature reviewing outcomes/complications and associated risk factors.**

| **Author**  **(year)**  **Country**  **Study design** | **Primary outcome measure (incidence)** | **Other outcomes** | **Risk factors and other relevant findings** |
| --- | --- | --- | --- |
| Furnaz et al.  (2023)  Pakistan | In-hospital mortality (6.7%) | Bacterial sepsis  (4.4%)  Surgical site infections (0.8%) | High risk procedures (RACHS-1 score 3), younger age (infancy./childhood), patients with SaO_2_ <85%, children with a genetic syndrome |
| Riley et al.  (2022)  USA  Paediatric cardiac surgery for CHD  (Scoping review) | Cardiac arrest  (3-6%) | Low occurrence of cardiac arrest results in other metrics of poor outcome (e.g. renal failure, death, emergency sternal opening) being used to power the analysis | Patient variables ass with outcome – younger age, lower weight, prematurity, genetic syndrome, PHT, extra-cardiac anomaly, preop nutritional status & mechanical ventilation  Surgical variable – increased surgical complexity (higher categories of RACHS-1 esp 4 & 5), longer CPB duration  Clinical variables – elevated postop lactate (delta lactate ≥2), lower median central venous oxygen saturations, higher median AVCO2, LCOS score  Other variable – 24/7 availability of ICU attending, nursing experience, reduced ICU staffing/hospital resources during weekends |
| Zheng et al.  (2021)  China  Children < 14 yrs of age for cardiac surgery  (retrospective) | Overall in hospital mortality (2.3%) |  | Lower age and weight; male gender; high Aristotle basic score; longer CPB, AXC and circulatory arrest time; and univentricular physiology were risk factors for in-hospital mortality |
| Zeng et al.  (2020)  China  Children < 14 yrs of age for cardiac surgery for CHD  (retrospective) | Identify risk factors associated with postop complications (29.3%), develop risk prediction tool for postop complications, and compare new tool to other prediction tools. Mortality rate found to be 5.5%. |  | Postop complications documented as cardiac (dysfunction resulting in low cardiac output, pulmonary hypertension, pericardial effusion, post procedural endocarditis, arrest), arythmias, neurologic deficit or paralysis of diaphragm, pulmonary (pneumonia, effusion, pneumothorax, insufficiency), renal (dysfunction requiring dialysis), infection (sepsis), wound infection and operative/procedural associated complications.  Risk factors associated with complications young; low weight and height; low blood oxygen saturation; longer surgical, CPB and AXC time; delayed sternal closure; emergency procedure multiple cardiac defects and multiple surgical procedures during admission. |
| Brown et al.  (2019)  UK  Children < 17 yrs having open, closed or hybrid surgery for CHD  (prospective) | Early postop morbidities (21.8%) | Short-term mortality (1.3%) | Early postop morbidities defined as 1 of the following 9: ECLS, prolonged pleural effusion, surgical infection, NEC, unplanned reoperation, feeding problems, RRT and major adverse events.  Major events = cardiac arrest, emergency chest reopening, major haemorrhage in CIU, never events (critical events such as wrong admin of medication transfusion, misplaced tubes, tissue or limb injury).  Risk factors were subcategorized and selected based on previous empirical data: age, gender, weight-for-age z score, complexity of cardiac lesion and surgery, CPD time, congenital/acquired comorbidity, Downs syndrome prematurity and severity of illness indicators (includes preop mechanical ventilation and shock). |
| Murni et al.  (2019)  Indonesia  Paediatric cardiac surgery for CHD  (prospective) | Major complications (19%) | Postop complications (84.1%) | Cyanotic CHD, longer CPB time, high dose of inotropes and increase in lactate associated with major complications (defined as presence of cardiac arrest, need for emergency relook and multi-organ failure). |
| Berger et al.  (2017)  USA  TOPICC database  (physiological profiles and development of functional morbidity)  (retrospective) | Comparison of the performance of FSS, PRISM (2 and 3 level) vs RACHS-1 and STAT complexity categories | Overall mortality 3.2% and new functional morbidity rate 4.8% (range 1.7-12.9% dependent on RACHS-1 or STAT category) | Adding RACHS-1 and STAT to the physiology based PRISM score didn’t improve model performance, indicating that the PRISM score had most of the information about surgical complexity, implication that physiological data needs to be considered and just the type/complexity of surgery. |
| Ross et al.  (2017)  USA  Children < 5 yrs presenting for cardiac surgery for CHD  (retrospective) | Impact of malnutrition on surgical outcomes | Mortality 2.3% | Low height-for-age and weight-for-age z-scores were associated with an increased risk of cardiac arrest, infection, mechanical ventilation, PLOS.  Age, gestational age at birth, weight, height, height-for-age z-score, weight-for-age z-score, major non-cardiac anomaly, CPB duration, duration of preop mechanical ventilation, RACHS-1 category 5-6, and defined genetic syndromes were associated with mortality. |
| Jacobs et al.  (2013)  USA  STSCHS database  (retrospective) | Major complications (1% for simple procedures-38.2% for TA with interrupted aortic arch repair vs 3.2% STAT category 1-30% stat category 5) | PLOS (2.9 – 42.6 days) | Empirically developed tool for estimating morbidity based on the procedure complexity and not individual patient risk factors.  Major complications defined as the occurrence of 1 or more of the following: postop ARF, neurologic deficit occurring even at discharge, AV block needing permanent pacemaker, mechanical circulatory support, phrenic nerve injury, unplanned reoperation. |
| Mattos et al.  (2006)  Brazil  Children ≤16 yrs of age, for cardiac surgery  (retrospective) | In hospital mortality (14.7%) based on index developed 5 variables | Index to predict mortality based on 5 variables (age, nutritional status, clinical risk factors, surgery complex as per RACHS-1 and ABC; CPB time) | Surgery in the neonatal phase, weight below the 5^th^ percentile, associated clinical risk factors (PHT, heart failure, cyanosis, acidosis, infection, genetic syndrome, non-elective mechanical ventilation and preop LOS), increased surgical complexity and CPB duration > 90 mins were associated with higher mortality. Other factors that had been identified prior to this include age, associated syndromes, phenotype of cardiac malformation, cardiac failure, AVSO2, CPB time, levels of glucose, lactate and CK, MAP and body tempt. |

*Murni included in Riley scoping review.

AVCO2 - arteriovenous CO2 differences, VIS – vasoactive infusion scores (total vasoactive requirements in patient on multiple vasoactive agents), LCOS – low cardiac output syndrome, PLOS – postoperative length of stay, FSS – Functional Status Scale (uses 6 domains mental status, sensory, communication, motor function, feeding, and respiratory each scored at 1 which is normal to 5 indicating severe dysfunction), decline in the FSS score ≥ 3 point from preop baseline to time of discharge is seen as a new morbidity.CPB – cardiopulmonary bypass, AVSO2 – arterial venous saturations of oxygen, CK- creatine kinase, MAP – mean arterial pressu
