# Supplementary material for: Risk factors associated with in-hospital mortality and a composite of major complications following paediatric congenital cardiac surgery: A systematic review protocol
Source: PLoS One. 2026 Nov 12;21(11):e0360693. doi: 10.1371/journal.pone.0360693 (PMC13759102; doi:10.1371/journal.pone.0360693)
Supplement: Supplementary file 2 [file pone.0360693.s003.docx]

RESPONSE TO REVIEWERS

**Risk factors associated with in-hospital mortality and a composite of major complications following paediatric congenital cardiac surgery: a systematic review protocol**

We thank the reviewers for their careful and constructive assessment of the manuscript. We have revised the protocol substantially to improve its methodological clarity, structure, statistical plan, definition of outcomes, rationale for the composite endpoint, citation practice, and language. The responses below address each comment individually.

**Editors Comments**

| Comments | Responses/ Corrections |
| --- | --- |
| 1. Manuscript must meet PLOS ONE’s style requirements. | Corrected. |
| 2. Supporting tables to be uploaded as separate folder labelled “Supporting Information”. | Corrected. |
| 3. Completing of data availability statement of submission form – prefer open data policy. | Corrected. |
| 4. Include full ethics statement in the ‘Methods’ and whether written or verbal consent were obtained. | Corrected. Line 194-196. |
| 5. If reviewer comments include recommendations to cite specific previously published works, pls review and evaluate to determine whether they are relevant and should be cited. | Comment made under reviewer 3 comments/corrections. |

**Comments to the Author**

| Comments | Responses/ Corrections |
| --- | --- |
| 1. Does the manuscript provide a valid rationale for the proposed study, with clearly identified and justified research questions? Partially | Changes made in introduction to address, particularly lines 152-166. |
| 2. Is the protocol technically sound and planned in a manner that will lead to a meaningful outcome and allow testing the stated hypotheses? Partially | Major revisions undertaken throughout document. |
| 3. Is the methodology feasible and described in sufficient detail to allow the work to be replicable? No | Methodology section rewritten as per comments made below. |
| 4. Have the authors described where all data underlying the findings will be made available when the study is complete? | All data related to findings will be made fully available without restriction. |
| 5. Is the manuscript presented in an intelligible fashion and written in standard English? | Language editing has taken assisted by various AI tools (ChatGP and Grammarly). |

**Reviewer 1**

| Comments | Responses/ Corrections |
| --- | --- |
| Guidelines of a systematic review have not been followed completely. Organization of contents under appropriate sections is incomplete. Abstract is written in a haphazard way. Authors are suggested to go through the guidelines and rewrite the article diligently. Grammatical and language errors need correction. | The manuscript has been comprehensively revised and reorganised in accordance with PRISMA-P principles. The abstract and main text have been restructured, the eligibility criteria and outcomes have been clarified, the risk-of-bias tools are now explicitly linked to study designs, and the synthesis strategy now specifies assessment of heterogeneity, conditions for meta-analysis, random-effects modelling, narrative synthesis where pooling is inappropriate, and assessment of publication bias where sufficient studies are available.  The manuscript has undergone comprehensive grammatical and language editing. |
|  |  |

**Reviewer 2**

| Comments | Responses/ Corrections |
| --- | --- |
| 1. Major Methodological Flaws The rationale (lines 36–38) heavily emphasizes the massive disparity in LMICs (>90% lack access; 10–12% mortality rates). However, the search strategy (lines 43–52) does not limit or target LMICs. | Abstract has been restructured and this information is discussed in background section.  The objective is to identify risk factors across paediatric congenital cardiac surgery populations globally, while comparing the applicability of the evidence to LMIC vs HIC settings. Restricting the search exclusively to LMICs could exclude important evidence used to establish robust risk factors. Where data permit, study setting and World Bank income classification will be extracted and considered in subgroup or stratified analyses.  Supporting information – Table 4 |
| 2. Inadequate Outcome & Risk Factor Definitions:The authors aim to identify risk factors for "a composite of major complications" (line 40), but fails to define what constitutes a major complication (e.g., re-operation, prolonged ventilation, renal replacement therapy, stroke). | The composite outcome has now been explicitly defined. It includes clinically important complications representing severe cardiovascular, neurological, renal, respiratory and surgical morbidity: cardiac arrest; low cardiac output syndrome; extracorporeal life support/mechanical circulatory support; prolonged pleural effusion; emergency reoperation; major haemorrhage requiring surgical re-exploration; multiorgan failure requiring organ support, including renal failure requiring dialysis; atrioventricular block requiring permanent pacemaker implantation; and new-onset neurological dysfunction persisting at discharge. The protocol also acknowledges that definitions vary between studies and prespecifies extraction of the original study definition for each component.  Line 112-122 Reason behind choosing specific composite of major complications.  Line 124-131 Definition thereof. |
| 3. Incomplete Risk of Bias Strategy: The authors list both the Newcastle-Ottawa Scale (NOS) and ROBINS-I (line 52). NOS and ROBINS-I assess non-randomized studies very differently, using distinct scoring paradigms and domains. The protocol must explicitly state which tool will be used for which study design (e.g., ROBINS-I for non-randomized intervention studies vs. NOS for observational cohort/case-control studies). | This has been clarified. ROBINS-I will be used for non-randomised studies of interventions where the risk-of-bias framework is applicable, while the Newcastle–Ottawa Scale will be used for observational cohort and case-control studies when appropriate. Study design will be established a priori during study selection, and the selected tool will be documented for each included study.  Line 270-278.  Line 284-287. |
| 4. Missing Synthesis & Analytical Strategy: There is no mention of:  1) How heterogeneity will be assessed  2) Whether a meta-analysis (random-effects vs. fixed-effects model) or a narrative synthesis (SWIM guidelines) will be conducted.  3) How publication bias will be evaluated (e.g., funnel plots, Egger's test). A review protocol cannot be evaluated or pre-registered without a clear meta-analytic plan. | The statistical analysis plan has been expanded.   1) Line 321-328   Clinical and methodological heterogeneity will first be assessed by comparing population characteristics, congenital diagnoses, surgical complexity, exposure definitions, outcome definitions, study design and adjustment strategies.   2) Line 309-319   Where sufficiently homogeneous studies report comparable effect estimates, quantitative synthesis will be undertaken using a random-effects model. Statistical heterogeneity will be assessed using I². Where meta-analysis is inappropriate because of substantial heterogeneity, incompatible outcome definitions or insufficient data, findings will be synthesised narratively in accordance with SWiM principles.   3) Line 332-339   Funnel plots and Egger's test will be considered when a sufficient number of studies are available (generally ≥10) to provide meaningful assessment of small-study effects/publication bias. |
| 5.Typographical Error in Database Names: Line 50 contains a noticeable typo: "Pudmed" instead of PubMed. | Corrected throughout manuscript. |
| 6. The authors should specify whether they are investigating pre-operative risk factors (e.g., nutritional status, age, genetic syndromes) or intra-operative/post-operative risk factors (e.g., cardiopulmonary bypass time, cross-clamp time). Grouping these together without a clear hierarchical model will confound the analysis. | Line 292-298  Risk factors are now explicitly categorised a priori into temporal domains: (1) pre-operative/patient-related factors, such as age, weight, prematurity, nutritional status, diagnosis, anatomical complexity and comorbidities; (2) intraoperative/surgical and anaesthetic factors, such as cardiopulmonary bypass and cross-clamp duration, operative variables and anaesthetic factors; and (3) postoperative/clinical factors, where relevant. These domains will be extracted separately and interpreted according to their temporal relationship with the outcome  p…p  line…. |
|  |  |
|  |  |

**Reviewer 3**

| Comments | Responses/ Corrections |
| --- | --- |
| 1. The rationale and novelty of the review should be strengthened by clearly  explaining how this study differs from previous reviews or existing evidence. | The rationale has been strengthened to distinguish this review from previous work. Existing literature includes studies examining individual complications, individual risk factors, selected patient populations, or specific outcomes, whereas this protocol proposes a systematic synthesis of risk factors associated with both in-hospital mortality and a predefined composite of major complications following paediatric congenital cardiac surgery. The review will also explicitly examine differences across study settings, including LMICs, and will address the heterogeneity in definitions of major morbidity. |
| 2. The authors should provide more details about the planned statistical analysis, including methods for handling heterogeneity. | The statistical analysis section has been expanded. See comment above on addressing heterogeneity. |
| 3. The authors should avoid citing many references together in a single sentence. | Line 358-371  The citation style has been revised throughout the manuscript. Individual references are now linked to the specific claims they support, with large clusters of unrelated citations removed. This is particularly evident in the sections describing mortality risk factors, major complications, and the rationale for the composite outcome. |
| 4. The search strategy should be expanded by including additional relevant databases and providing the complete search strings for all databases. | See Supporting Information Table 3.  The revised protocol specifies the databases to be searched and provides the complete electronic search strategy for each database, including controlled vocabulary and free-text terms. The search strategy will be adapted appropriately for the indexing structure of each database rather than using an identical string across platforms. |
| 5. Authors should cite the provided literature as it was relevant of the Reviewer. The manuscript should avoid bulk citations. Provide a clear cluster in the introduction and cite the work in reference lists.  a) <https://doi.org/10.1016/j.biopha.2020.110525>  b) 10.2147/DDDT.S282206 c) <https://doi.org/10.1016/j.biopha.2024.116337>  d) <https://doi.org/10.2147/JIR.S502203>  e) 10.3389/fphar.2025.1679085 f) [https://doi.org/10.1073/pnas.2317801121](https://doi.org/10.1073/pnas.2317801121) g) [https://doi.org/10.1161/JAHA.125.043867](https://doi.org/10.1161/JAHA.125.043867) | The suggested references have been considered, however we found that it did not fit this systematic review’s eligible primary evidence, rather the studies support a biological/inflammatory/  metabolic rationale.  a) Xu, NF-KB in rat model  b) Jiang, xanthohumol, TGF-B1  c) Deng, mitophagy in MI and reperfusion injury  d)Zhu, necroptosis and immune infiltration in heart failure  e) Wang, mitochondrial dysfunction  f) Shi, animal model GATA6 hepatic disease  g) Huang, environmental factors and stroke |
| 6. The manuscript should better define the composite outcome of major complications and explain the rationale for selecting these specific complications | Addressed as above (reviewer 2 comment). |
| 7. The limitations of the proposed systematic review, including language restriction, potential publication bias, and variability among included studies, should be discussed more clearly. | Line 382-395.  A dedicated limitations paragraph has been added. |
| 8. The manuscript requires careful language editing to correct grammatical and terminology errors, including “cardioembolic risk markers,” which should likely be “cardiometabolic risk markers.” | The terminology has been corrected to 'cardiometabolic risk markers'. The manuscript has also undergone comprehensive language editing to address grammar, spelling, punctuation, terminology, consistency and readability. |
|  |  |

Closing statement

We are grateful to all three reviewers for their constructive comments. The revisions have strengthened the protocol by improving methodological transparency, clarifying the definition and rationale for the composite outcome, strengthening the statistical synthesis plan, improving the organisation and reporting of the protocol, and addressing citation and language concerns.
