# Supplementary material for: Risk factors associated with in-hospital mortality and a composite of major complications following paediatric congenital cardiac surgery: A systematic review protocol
Source: PLoS One. 2026 Nov 12;21(11):e0360693. doi: 10.1371/journal.pone.0360693 (PMC13759102; doi:10.1371/journal.pone.0360693)
Supplement: Peer review history

# Peer review: Decision Letter 0

**DOI:** 10.1371/journal.pone.0360693.r001

**Authors:** Eyüp Serhat Çalık

5 Aug 2026

Dear Dr. Mogane,

Thank you for submitting your manuscript to PLOS ONE. After careful consideration, we feel that it has merit but does not fully meet PLOS ONE’s publication criteria as it currently stands. Therefore, we invite you to submit a revised version of the manuscript that addresses the points raised during the review process.

Please submit your revised manuscript by Sep 04 2026 11:59PM. If you will need more time than this to complete your revisions, please reply to this message or contact the journal office at plosone@plos.org. When you're ready to submit your revision, log on to https://www.editorialmanager.com/pone/ and select the 'Submissions Needing Revision' folder to locate your manuscript file.

- A letter that responds to each point raised by the academic editor and reviewer(s). You should upload this letter as a separate file labeled 'Response to Reviewers'.
- A marked-up copy of your manuscript that highlights changes made to the original version. You should upload this as a separate file labeled 'Revised Manuscript with Track Changes'.
- An unmarked version of your revised paper without tracked changes. You should upload this as a separate file labeled 'Manuscript'.

If you would like to make changes to your financial disclosure, please include your updated statement in your cover letter. Guidelines for resubmitting your figure files are available below the reviewer comments at the end of this letter.

If applicable, we recommend that you deposit your laboratory protocols in protocols.io to enhance the reproducibility of your results. Protocols.io assigns your protocol its own identifier (DOI) so that it can be cited independently in the future. For instructions see: https://journals.plos.org/plosone/s/submission-guidelines#loc-laboratory-protocols. Additionally, PLOS ONE offers an option for publishing peer-reviewed Lab Protocol articles, which describe protocols hosted on protocols.io. Read more information on sharing protocols at https://plos.org/protocols?utm_medium=editorial-email&utm_source=authorletters&utm_campaign=protocols.

As the corresponding author, your ORCID iD is verified in the submission system and will appear in the published article. PLOS supports the use of ORCID, and we encourage all coauthors to register for an ORCID iD and use it as well. Please encourage your coauthors to verify their ORCID iD within the submission system before final acceptance, as unverified ORCID iDs will not appear in the published article. *Only*  the individual author can complete the verification step; PLOS staff *cannot*  verify ORCID iDs on behalf of authors.

We look forward to receiving your revised manuscript.

Kind regards,

Eyüp Serhat Çalık

Academic Editor

PLOS One

Journal Requirements:

When submitting your revision, we need you to address these additional requirements.

1. Please ensure that your manuscript meets PLOS ONE's style requirements, including those for file naming. The PLOS ONE style templates can be found at

https://journals.plos.org/plosone/s/file?id=wjVg/PLOSOne_formatting_sample_main_body.pdf and

https://journals.plos.org/plosone/s/file?id=ba62/PLOSOne_formatting_sample_title_authors_affiliations.pdf

2. We notice that your supplementary tables are included in the manuscript file. Please remove them and upload them with the file type 'Supporting Information'. Please ensure that each Supporting Information file has a legend listed in the manuscript after the references list.

3. When completing the data availability statement of the submission form, you indicated that you will make your data available on acceptance. We strongly recommend all authors decide on a data sharing plan before acceptance, as the process can be lengthy and hold up publication timelines. Please note that, though access restrictions are acceptable now, your entire data will need to be made freely accessible if your manuscript is accepted for publication. This policy applies to all data except where public deposition would breach compliance with the protocol approved by your research ethics board. If you are unable to adhere to our open data policy, please kindly revise your statement to explain your reasoning and we will seek the editor's input on an exemption. Please be assured that, once you have provided your new statement, the assessment of your exemption will not hold up the peer review process.

4. Please include your full ethics statement in the ‘Methods’ section of your manuscript file. In your statement, please include the full name of the IRB or ethics committee who approved or waived your study, as well as whether or not you obtained informed written or verbal consent. If consent was waived for your study, please include this information in your statement as well.

5. If the reviewer comments include a recommendation to cite specific previously published works, please review and evaluate these publications to determine whether they are relevant and should be cited. There is no requirement to cite these works unless the editor has indicated otherwise.

Additional Editor Comments:

I congratulate the authors on this important study they have planned. They present a clinically significant and relevant systematic review protocol focusing on risk factors associated with in-hospital mortality and major complications following pediatric congenital heart surgery. While the methodology is generally sound, certain methodological details regarding the search strategy, outcome definitions, statistical synthesis methods, and the handling of heterogeneity require further clarification.

The manuscript was reviewed by three external reviewers; their recommendations are listed below. We look forward to you re-uploading your manuscript after making the necessary revisions based on these recommendations and providing appropriate, point-by-point answers to the questions raised. Best of luck.

[Note: HTML markup is below. Please do not edit.]

Reviewers' comments:

Reviewer's Responses to Questions

**Comments to the Author**

1. Does the manuscript provide a valid rationale for the proposed study, with clearly identified and justified research questions?

Reviewer #1: Partly

Reviewer #2: Partly

Reviewer #3: Yes

**********

2. Is the protocol technically sound and planned in a manner that will lead to a meaningful outcome and allow testing the stated hypotheses?

Reviewer #1: Partly

Reviewer #2: No

Reviewer #3: Yes

**********

3. Is the methodology feasible and described in sufficient detail to allow the work to be replicable?

Reviewer #1: No

Reviewer #2: No

Reviewer #3: Yes

**********

4. Have the authors described where all data underlying the findings will be made available when the study is complete?

The PLOS Data policy

Reviewer #1: Yes

Reviewer #2: No

Reviewer #3: Yes

**********

5. Is the manuscript presented in an intelligible fashion and written in standard English?

Reviewer #1: No

Reviewer #2: No

Reviewer #3: Yes

**********

Please use the space provided to explain your answers to the questions above and, if applicable, provide comments about issues authors must address before this protocol can be accepted for publication. You may also include additional comments for the author, including concerns about research or publication ethics.

You may also provide optional suggestions and comments to authors that they might find helpful in planning their study.

Reviewer #1: Dear authors,

I have reviewed the article tilted, "Risk factors associated with in-hospital mortality and a composite of major complications following paediatric congenital cardiac surgery: a systematic review protocol".

I would appreciate authors efforts but guidelines of a systematic review has not been followed completely. Organization of contents under appropriate sections is incomplete. Abstract is written in a haphazard way. Authors are suggested to go through the guidelines and rewrite the article deligently. Grammatical and language errors need correction.

Reviewer #2: The protocol outlines a systematic review aimed at identifying risk factors for in-hospital mortality and major complications in pediatric patients undergoing congenital heart surgery, with a particular emphasis on low- and middle-income countries (LMICs). While the clinical problem is critically important, the current proposal suffers from significant methodological gaps, structural inconsistencies, and draft-level formatting issues. Moreover, I offer the following comments:

1. Major Methodological Flaws

The rationale (lines 36–38) heavily emphasizes the massive disparity in LMICs (>90% lack access; 10–12% mortality rates). However, the search strategy (lines 43–52) does not limit or target LMICs.

2. Inadequate Outcome & Risk Factor Definitions:

The authors aim to identify risk factors for "a composite of major complications" (line 40), but fails to define what constitutes a major complication (e.g., re-operation, prolonged ventilation, renal replacement therapy, stroke).

3. Incomplete Risk of Bias Strategy:

The authors list both the Newcastle-Ottawa Scale (NOS) and ROBINS-I (line 52). NOS and ROBINS-I assess non-randomized studies very differently, using distinct scoring paradigms and domains. The protocol must explicitly state which tool will be used for which study design (e.g., ROBINS-I for non-randomized intervention studies vs. NOS for observational cohort/case-control studies).

4. Missing Synthesis & Analytical Strategy:

There is no mention of:

1) How heterogeneity will be assessed

2) Whether a meta-analysis (random-effects vs. fixed-effects model) or a narrative synthesis (SWIM guidelines) will be conducted.

3) How publication bias will be evaluated (e.g., funnel plots, Egger's test).

A review protocol cannot be evaluated or pre-registered without a clear meta-analytic plan.

5.Typographical Error in Database Names:

Line 50 contains a noticeable typo: "Pudmed" instead of PubMed.

6. The authors should specify whether they are investigating pre-operative risk factors (e.g., nutritional status, age, genetic syndromes) or intra-operative/post-operative risk factors (e.g., cardiopulmonary bypass time, cross-clamp time). Grouping these together without a clear hierarchical model will confound the analysis.

Reviewer #3: PLOS One

Risk factors associated with in-hospital mortality and a composite of major complications following paediatric congenital cardiac surgery: a systematic review protocol

This manuscript presents a systematic review protocol aimed at identifying risk factors associated with in-hospital mortality and major complications following paediatric congenital cardiac surgery. The study plans to analyse evidence from published cohort, case-control, and registry studies to determine patient, surgical, anaesthetic, and clinical factors influencing outcomes. The authors will perform a comprehensive literature search, assess study quality, and synthesize available data using established systematic review methodologies. The findings are expected to improve risk assessment, perioperative management, and decision-making for children undergoing congenital heart surgery. The following issues are to be addressed very neatly and precisely.

1. The rationale and novelty of the review should be strengthened by clearly explaining how this study differs from previous reviews or existing evidence.

2. The authors should provide more details about the planned statistical analysis, including methods for handling heterogeneity.

3. The authors should avoid citing many references together in a single sentence.

4. The search strategy should be expanded by including additional relevant databases and providing the complete search strings for all databases.

5. Authors should cite the provided literature as it was relevant of the Reviewer. The manuscript should avoid bulk citations. Provide a clear cluster in the introduction and cite the work in reference lists.

a) https://doi.org/10.1016/j.biopha.2020.110525 b) 10.2147/DDDT.S282206

c) https://doi.org/10.1016/j.biopha.2024.116337 d) https://doi.org/10.2147/JIR.S502203

e) 10.3389/fphar.2025.1679085 f) https://doi.org/10.1073/pnas.2317801121

g) https://doi.org/10.1161/JAHA.125.043867

6. The manuscript should better define the composite outcome of major complications and explain the rationale for selecting these specific complications

7. The limitations of the proposed systematic review, including language restriction, potential publication bias, and variability among included studies, should be discussed more clearly.

8. The manuscript requires careful language editing to correct grammatical and terminology errors, including “cardioembolic risk markers,” which should likely be “cardiometabolic risk markers.”

**********

what does this mean?). If published, this will include your full peer review and any attached files.

If you choose “no”, your identity will remain anonymous but your review may still be made public.

**Do you want your identity to be public for this peer review?**  For information about this choice, including consent withdrawal, please see our Privacy Policy

Reviewer #1: No

Reviewer #2: No

Reviewer #3: No

**********

[NOTE: If reviewer comments were submitted as an attachment file, they will be attached to this email and accessible via the submission site. Please log into your account, locate the manuscript record, and check for the action link "View Attachments". If this link does not appear, there are no attachment files.]

To ensure your figures meet our technical requirements, please review our figure guidelines: https://journals.plos.org/plosone/s/figures

You may also use PLOS’s free figure tool, NAAS, to help you prepare publication quality figures: https://journals.plos.org/plosone/s/figures#loc-tools-for-figure-preparation.

NAAS will assess whether your figures meet our technical requirements by comparing each figure against our figure specifications.

# Author comment: Author response to Decision Letter 1

**DOI:** 10.1371/journal.pone.0360693.r002

5 Sep 2026

Please find attached detailed letter addressing comments and concerns made by the reviewers.

Attachment Submitted filename: Response to reviewers PONE 5 Sept.docx

# Peer review: Decision Letter 1

**DOI:** 10.1371/journal.pone.0360693.r003

**Authors:** Eyüp Serhat Çalık

29 Sep 2026

Risk factors associated with in-hospital mortality and a composite of major complications following paediatric congenital cardiac surgery: a systematic review protocol

PONE-D-26-17813R1

Dear Dr. Mogane,

We’re pleased to inform you that your manuscript has been judged scientifically suitable for publication and will be formally accepted for publication once it meets all outstanding technical requirements.

Within one week, you’ll receive an e-mail detailing the required amendments. When these have been addressed, you’ll receive a formal acceptance letter and your manuscript will be scheduled for publication.

An invoice will be generated when your article is formally accepted. Please note, if your institution has a publishing partnership with PLOS and your article meets the relevant criteria, all or part of your publication costs will be covered. Please make sure your user information is up-to-date by logging into Editorial Manager at Editorial Manager® and clicking the ‘Update My Information' link at the top of the page. For questions related to billing, please contact billing support.

If your institution or institutions have a press office, please notify them about your upcoming paper to help maximize its impact. If they’ll be preparing press materials, please inform our press team as soon as possible -- no later than 48 hours after receiving the formal acceptance. Your manuscript will remain under strict press embargo until 2 pm Eastern Time on the date of publication. For more information, please contact onepress@plos.org.

Kind regards,

Eyüp Serhat Çalık

Academic Editor

PLOS One

Additional Editor Comments (optional):

Reviewers' comments:

Reviewer's Responses to Questions

**Comments to the Author**

1. Does the manuscript provide a valid rationale for the proposed study, with clearly identified and justified research questions?

Reviewer #2: Yes

Reviewer #3: Yes

**********

2. Is the protocol technically sound and planned in a manner that will lead to a meaningful outcome and allow testing the stated hypotheses?

Reviewer #2: Yes

Reviewer #3: Yes

**********

3. Is the methodology feasible and described in sufficient detail to allow the work to be replicable?

Reviewer #2: Yes

Reviewer #3: Yes

**********

4. Have the authors described where all data underlying the findings will be made available when the study is complete?

The PLOS Data policy

Reviewer #2: Yes

Reviewer #3: No

**********

5. Is the manuscript presented in an intelligible fashion and written in standard English?

Reviewer #2: Yes

Reviewer #3: Yes

**********

Please use the space provided to explain your answers to the questions above and, if applicable, provide comments about issues authors must address before this protocol can be accepted for publication. You may also include additional comments for the author, including concerns about research or publication ethics.

You may also provide optional suggestions and comments to authors that they might find helpful in planning their study.

Reviewer #2: The manuscript is of high scholarly merit and addresses a compelling research question. I thoroughly enjoyed examining this work, and its findings are robust enough to warrant wide dissemination.

Reviewer #3: The authors have responded to all raised concerns. I accept it in the current form. The revisions adequately address the points brought up during the review process. The clarifications provided in the response letter and the corresponding changes made in the manuscript have improved the quality of the paper. Therefore, I recommend acceptance.

**********

what does this mean?). If published, this will include your full peer review and any attached files.

If you choose “no”, your identity will remain anonymous but your review may still be made public.

**Do you want your identity to be public for this peer review?**  For information about this choice, including consent withdrawal, please see our Privacy Policy

Reviewer #2: No

Reviewer #3: No

**********

# Editor report: Acceptance letter

**DOI:** 10.1371/journal.pone.0360693.r004

**Authors:** Eyüp Serhat Çalık

PONE-D-26-17813R1

PLOS One

Dear Dr. Mogane,

I'm pleased to inform you that your manuscript has been deemed suitable for publication in PLOS One. Congratulations! Your manuscript is now being handed over to our production team.

At this stage, our production department will prepare your paper for publication. This includes ensuring the following:

* All references, tables, and figures are properly cited

* All relevant supporting information is included in the manuscript submission,

* There are no issues that prevent the paper from being properly typeset

You will receive further instructions from the production team, including instructions on how to review your proof when it is ready. Please keep in mind that we are working through a large volume of accepted articles, so please give us a few days to review your paper and let you know the next and final steps.

Lastly, if your institution or institutions have a press office, please let them know about your upcoming paper now to help maximize its impact. If they'll be preparing press materials, please inform our press team within the next 48 hours. Your manuscript will remain under strict press embargo until 2 pm Eastern Time on the date of publication. For more information, please contact onepress@plos.org.

You will receive an invoice from PLOS for your publication fee after your manuscript has reached the completed accept phase. If you receive an email requesting payment before acceptance or for any other service, this may be a phishing scheme. Learn how to identify phishing emails and protect your accounts at https://explore.plos.org/phishing.

If we can help with anything else, please email us at customercare@plos.org.

Thank you for submitting your work to PLOS One and supporting open access.

Kind regards,

PLOS One Editorial Office Staff

on behalf of

Dr. Eyüp Serhat Çalık

Academic Editor

PLOS One
